# Mental Health–Related Outpatient Visits Among Adolescents and Young Adults, 2006-2019

**DOI:** 10.1001/jamanetworkopen.2024.1468

**Published:** 2024-03-07

**Authors:** Rosa Y. Ahn-Horst, Florence T. Bourgeois

**Affiliations:** 1 Department of Psychiatry, Massachusetts General Hospital, Boston; 2 Department of Psychiatry, McLean Hospital, Belmont, Massachusetts; 3 Department of Psychiatry, Harvard Medical School, Boston, Massachusetts; 4 Computational Health Informatics Program, Boston Children’s Hospital, Boston, Massachusetts; 5 Department of Pediatrics, Harvard Medical School, Boston, Massachusetts

## Abstract

**Importance:**

Concerns over the mental health of young people have been increasing over the past decade, especially with the rise in mental health burden seen during the COVID-19 pandemic. Examining trends in mental health–related outpatient visits provides critical information to elucidate contributing factors, identify vulnerable populations, and inform strategies to address the mental health crisis.

**Objective:**

To examine characteristics and trends in mental health–related outpatient visits and psychotropic medication use among US adolescents and young adults.

**Design, Setting, and Participants:**

A retrospective cross-sectional analysis of nationally representative data from the National Ambulatory Medical Care Survey, an annual probability sample survey, was conducted from January 2006 to December 2019. Participants included adolescents (age 12-17 years) and young adults (age 18-24 years) with office-based outpatient visits in the US. Data were analyzed from March 1, 2023, to September 15, 2023.

**Main Outcomes and Measures:**

Mental health–related outpatient visits were identified based on established sets of diagnostic codes for psychiatric disorders. Temporal trends in the annual proportion of mental health–related outpatient visits were assessed, including visits associated with use of psychotropic medications. Analyses were stratified by age and sex.

**Results:**

From 2006 to 2019, there were an estimated 1.1 billion outpatient visits by adolescents and young adults, of which 145.0 million (13.1%) were associated with a mental health condition (mean [SD] age, 18.4 [3.5] years; 74.0 million females [51.0%]). Mental health–related diagnoses were more prevalent among visits by male (16.8%) compared with female (10.9%) patients (*P* < .001). This difference was most pronounced among young adults, with 20.1% of visits associated with a psychiatric diagnosis among males vs 10.1% among females (*P* < .001). The proportion of mental health–related visits nearly doubled, from 8.9% in 2006 to 16.9% in 2019 (*P* < .001). Among all outpatient visits, 17.2% were associated with the prescription of at least 1 psychotropic medication, with significant increases from 12.8% to 22.4% by 2019 (*P* < .001).

**Conclusions and Relevance:**

In this cross-sectional study, there were substantial increases in mental health–related outpatient visits and use of psychotropic medications, with greater overall burden among male patients. These findings provide a baseline for understanding post-pandemic shifts and suggest that current treatment and prevention strategies will need to address preexisting psychiatric needs in addition to the effects of the COVID-19 pandemic.

## Key Points

**Question:**

What are the trends in mental health–related outpatient visits and psychotropic medication use among adolescents and young adults in the US from 2006 to 2019?

**Findings:**

In this cross-sectional analysis of nationally representative data, the proportion of mental health–related outpatient visits and visits associated with psychotropic medications increased almost 2-fold. There were significant increases specifically for visits related to mood, behavioral conditions, and substance use.

**Meaning:**

The findings of this study suggest that youth experienced a significant and sustained increase in mental health burden for over a decade preceding the COVID-19 pandemic, and treatment and prevention strategies will need to address preexisting psychiatric needs in addition to the direct effects of the COVID-19 pandemic.

## Teaser

This cross-sectional study examines changes from 2006 to 2019 in mental health–related outpatient visits and psychotropic medication use among US adolescents and young adults.

## Introduction

Concerns over the mental health of young people have been increasing over the past decade. From 2008 to 2015, hospitalization for suicidal behaviors doubled, and an estimated 1 in every 5 children in the US experienced a mental illness.^1,2,3^ The COVID-19 pandemic further increased the burden of mental health illness in this population, with alarming increases in mental health–related emergency department visits and suicidal behaviors, particularly among female adolescents.^4^ In response, in 2019, the American Academy of Pediatrics declared a national state of emergency in children’s mental health and called for improved strategies to effectively address mental health needs.^5^ Examining trends in the prevalence of mental health conditions is necessary to address this crisis and understand contributing factors, identify vulnerable populations, and inform strategies to provide effective services.

Studies have documented an increase in emergency department visits by adolescents and young adults over the past decade,^6,7,8^ but it is unknown whether there has been a similar sustained increase in ambulatory visits in this population.^9,10^ Understanding trends in the diagnosis and treatment of mental health conditions in outpatient settings is critical as these health care encounters represent the most common avenue through which adolescents and young adults access mental health care. Most studies have traditionally focused solely on the pediatric or adult population, with few considering young adults (age 18-24 years) as a separate group, despite ample evidence that these individuals are unique in terms of clinical risk profiles and the emergence of psychiatric illnesses.^11,12,13,14^ In addition, sex-based analyses are imperative in assessing mental health conditions because of differences in prevalence, presentation, risk factors, and course.^15^ Understanding these differences can contribute to improved diagnosis, treatment, and prevention approaches for both sexes.

The objectives of this study were to examine characteristics and trends over time from 2006 to 2019 for mental health–related outpatient visits among adolescents and young adults, including the use of psychotropic medications.

## Methods

### Data Source and Sample

This study was a retrospective cross-sectional analysis of the National Ambulatory Medical Care Survey (NAMCS), from January 2006 to December 2019, exclusive of 2017, as data for this year have not been made available. The NAMCS, administered annually by the National Center for Health Statistics (NCHS) of the Centers for Disease Control and Prevention, is a national probability survey of visits to office-based physicians engaged in direct patient care.^16^ It uses a 3-stage probability sample design based on geography, physician practices within a geographic location, and visits within physician practices. Trained health care professionals complete patient record forms for patient visits, which is the unit of analysis. Each visit is weighted to allow for the calculation of national estimates. For this study, we identified visits for adolescents (age 13-17 years) and young adults (age 18-24 years). The survey response rate varied from 31.2% to 62.4% (median, 45% [IQR: 39%-59%]) over the 14-year period and was accounted for by sampling weights.^17^ The NAMCS was approved by the National Center for Health Statistics Research Ethics Review Board and did not require institutional review board approval at Massachusetts General Hospital and McLean Hospital as all data are deidentified. We followed the Strengthening the Reporting of Observational Studies in Epidemiology (STROBE) reporting guideline.

Mental health–related outpatient visits included visits with diagnoses for psychiatric or substance use disorders, which were identified based on *International Classification of Diseases, Ninth Revision, Clinical Modification* (*ICD-9-CM*) (2006-2015) and *International Statistical Classification of Diseases, Tenth Revision, Clinical Modification* (*ICD-10-CM*) (2016-2019) codes.^18,19^ Up to 3 diagnoses were recorded for each visit from 2006 to 2013, and up to 5 for each visit from 2014 to 2019. We limited the number of diagnoses to the first 3 for all years for consistency. Visits were included when any 1 of the 3 diagnosis codes was for a mental health condition.

Based on prior US-based studies examining the burden of mental health conditions, we classified psychiatric diagnoses into 6 categories: (1) mood-related (eg, depression, anxiety, bipolar disorder, trauma, and stress-related conditions), (2) behavioral (eg, disruptive, impulse-control, and attention-deficit/hyperactive disorders), (3) psychosis (eg, schizophrenia, schizoaffective, and delusional disorders), (4) suicide-related (eg, suicidal ideation, suicidal attempts, and nonsuicidal self-injury), (5) substance use, and (6) other (eg, tic disorders, eating disorders, and personality disorders) (eTable 1 in Supplement 1).^6,7^ Diagnosis codes for neurodevelopmental disorders, such as autism spectrum disorder, were not included, consistent with the classification of mental health conditions in the Centers for Disease Control and Prevention National Syndromic Surveillance Program.^20^

Medications associated with visits were grouped using the Multum therapeutics classification system. The NAMCS collected data on up to 8 medications from 2006 to 2011, up to 10 medications from 2012 to 2013, and up to 30 from 2013 to 2019. We limited the number of medications to the first 8 for consistency across all years. We identified all psychotropic medications associated with visits and categorized these into 1 of 7 drug classes: (1) antidepressants; (2) antipsychotics; (3) central nervous system stimulants; (4) anxiolytics, sedatives, and hypnotics; (5) mood stabilizers; (6) medications for substance use; and (7) antiadrenergic agents (eTable 2 in Supplement 1).^21^

Sociodemographic characteristics analyzed included age, sex, race and ethnicity (abstracted from electronic health records as non-Hispanic Black, Hispanic, non-Hispanic White, and non-Hispanic Other (American Indian or Alaska Native, Asian, Native Hawaiian/Other Pacific Islander, and multiple races), insurance type (private, public, self-pay, and other), geographic region (Northeast, South, West, and Southwest), and metropolitan statistical area status. Race and ethnicity were examined as these characteristics have been shown to be associated with variable use of health care services for mental health conditions.^22^

### Statistical Analysis

Descriptive statistics were used to describe visit characteristics and examine differences in the prevalence of mental health–related outpatient visits by sex and age (adolescents and young adults). We also determined the prevalence of mental health–related visits associated with at least 1 psychotropic medication by sex and age, with χ^2^ tests used for comparisons.

We assessed temporal trends in the proportion of mental health–related visits and outpatient visits associated with psychotropic medications using χ^2^ tests for linear trend. Trend analyses were conducted using annual proportions, but for presentation purposes, annual data were combined into 2-year periods.

Estimates based on less than 30 unweighted observations are considered unreliable by the NCHS and were flagged in the results.^23^ Analysis of suicide-related diagnoses was not possible as the total sample size consisted of less than 30 visits and did not support further stratification.

Analyses were conducted using Stata, version 18 (StataCorp LLC) from March 1, 2023, to September 15, 2023.^24^ The *svy* commands were used to produce national estimates to account for the multistage survey sample design, as recommended by the NCHS.^25^ The χ^2^ tests by default were 2-sided, and statistical significance was set at *P* < .05.

## Results

### Characteristics of Mental Health–Related Visits

From 2006 to 2019, there were an estimated 1.1 billion outpatient visits by adolescents and young adults, of which 145.0 million (13.1%) were associated with a mental health condition. Demographic characteristics of patients with mental health–related visits are reported in Table 1. Patients had a mean (SD) age of 18.4 (3.5) years, with similar representation of females (51.0%) and males (49.0%), although males made up a greater proportion of visits associated with mental health conditions compared with visits for non–mental health conditions (49.0% vs 36.7%; *P* < .001). Most patients were non-Hispanic White (77.0%), had private insurance (56.0%), and were based in metropolitan areas (89.2%). In addition to patient sex, race and ethnicity, insurance type, and geographic location differed between visits with and without mental health conditions.

**Table 1.** Characteristics of Outpatient Visits Among Adolescents and Young Adults, 2006-2019

Characteristic	No. (%)			*P* value
	All visits	Mental health–related visits	Non–mental health–related visits	
Sample size				
Unweighted total visits	37 714	5497	32 217	NA
Weighted total visits	1 105 023 336	145 004 533 (13.1)	960 018 803 (86.9)	
Age, mean (SD), y	18.4 (3.5)	18.5 (3.5)	18.4 (3.5)	>.99
Sex				
Female	681 918 700	73 976 580 (51.0)	607 942 120 (63.3)	<.001
Male	423 104 636	71 027 953 (49.0)	352 076 683 (36.7)	
Race and ethnicity				
Hispanic	146 114 339 (17.6)	13 187 566 (10.9)	132 926 773 (18.7)	<.001
Non-Hispanic Black	96 062 613 (11.5)	8 535 212 (7.7)	87 527 401 (12.3)	
Non-Hispanic White	548 952 038 (65.9)	93 299 363 (77.0)	455 652 675 (64.0)	
Non-Hispanic other^a^	41 549 949 (5.0)	6 114 197 (5.1)	35 435 752 (5.0)	
Insurance type				
Private	667 825 981 (60.4)	81 251 986 (56.0)	586 573 995 (61.1)	<.001
Public^b^	285 019 240 (25.8)	31 815 490 (21.9)	253 203 750 (26.4)	
Self-pay	67 699 382 (6.1)	20 727 309 (14.3)	46 972 073 (4.9)	
Other^c^	84 478 733 (7.6)	11 209 748 (7.7)	73 268 985 (7.6)	
Geographic region				
Northeast	186 873 901 (19.4)	28 991 682 (24.0)	157 882 219 (18.8)	.02
South	368 967 753 (38.4)	42 337 085 (35.1)	326 630 668 (38.9)	
Midwest	197 644 376 (20.6)	25 994 851 (21.5)	171 649 525 (20.4)	
West	207 600 516 (21.6)	23 374 108 (19.4)	184 226 408 (21.9)	
Metropolitan Statistical Area status	995 005 945 (90.0)	129 308 430 (89.2)	865 697 065 (90.2)	.45

Abbreviation: NA, not applicable.

^a^ Non-Hispanic other includes Asian, Native Hawaiian/Other Pacific Islander, American Indian or Alaska Native, and multiple races.

^b^ Public includes Medicare, Medicaid, and worker’s compensation.

^c^ Other includes no charge, unknown, and other.

Overall, mental health–related diagnoses were more prevalent among visits by male patients compared with female patients, with 16.8% of visits by males associated with a psychiatric diagnosis and 10.9% of visits by females (*P* < .001) (Table 2). This difference was most pronounced among young adults, where 20.1% of visits by male patients were associated with a psychiatric diagnosis compared with 10.1% of visits by female patients (*P* < .001).

**Table 2.** Mental Health–Related Outpatient Visits Among Adolescents and Young Adults by Sex

Variable	No. (%)			*P* value
	All visits (n = 1 105 023 336)	Visits by female patients (n = 681 918 700)	Visits by male patients (n = 423 104 636)	
Any psychiatric diagnosis, age	145 004 533 (13.1)	73 976 580 (10.9)	71 027 953 (16.8)	<.001
13-17 y	62 690 951 (13.0)	31 170 454 (12.6)	31 520 497 (13.9)	<.001
18-24 y	82 313 582 (13.2)	42 806 126 (10.1)	39 507 456 (20.1)	<.001
Mood, age	97 826 566 (8.9)	56 180 675 (8.2)	41 645 892 (9.8)	.003
13-17 y	37 718 785 (7.8)	22 629 082 (9.2)	15 089 703 (7.0)	.003
18-24 y	60 107 782 (9.7)	33 551 593 (7.9)	26 556 189 (13.5)	<.001
Behavioral, age	57 849 306 (5.2)	22 661 608 (3.3)	35 187 698 (8.3)	<.001
13-17 y	35 005 123 (7.2)	12 742 992 (5.0)	22 262 131 (9.8)	<.001
18-24 y	22 844 183 (3.7)	9 918 616 (2.3)	12 925 567 (6.6)	<.001
Psychosis, age	3 536 095 (0.32)	923 044 (0.14)	2 613 051 (0.62)	<.001
13-17 y	959 146 (0.20)	354 458 (0.14)^a^	604 687 (0.27)^a^	.44
18-24 y	2 576 949 (0.41)	568 585 (0.13)	2 008 364 (1)	<.001
Substance use, age	14 326 800 (1.3)	5 847 244 (0.86)	8 479 556 (2.0)	<.001
13-17 y	2 430 244 (0.50)	1 359 087 (0.53)	1 071 157 (0.47)	.76
18-24 y	11 896 556 (1.9)	4 488 156 (1.1)	7 408 400 (3.8)	<.001
Other, age	5 502 481 (0.50)	3 521 363 (0.52)	1 981 118 (0.47)	.25
13-17 y	2 369 640 (0.49)	1 458 002 (0.57)	911 638 (0.40)	.25
18-24 y	3 132 841 (0.50)	2 063 361 (0.48)	1 069 480 (0.54)	.63

^a^ Per the National Center for Health Statistics, these estimates are considered unreliable since they are based on a sample size of less than 30 visits.

The most common categories of psychiatric conditions were mood-related (8.9%) and behavioral (5.2%). Among visits by adolescents, those by female patients were more likely to be associated with a mood disorder (9.2% vs 7.0%; *P* = .003). However, this trend reversed for visits by young adults, with 13.5% of visits by male patients associated with a mood disorder compared with 7.9% of visits by female patients (*P* < .001). Behavioral conditions were more common among visits by male patients in both age groups, with nearly twice the prevalence among visits by adolescents (5.0% vs 9.8%; *P* < .001), and nearly triple the prevalence among visits by young adults (2.3% vs 6.6%; *P* < .001).

### Trends in Visits for Mental Health–Related Conditions

**Figure 1.**
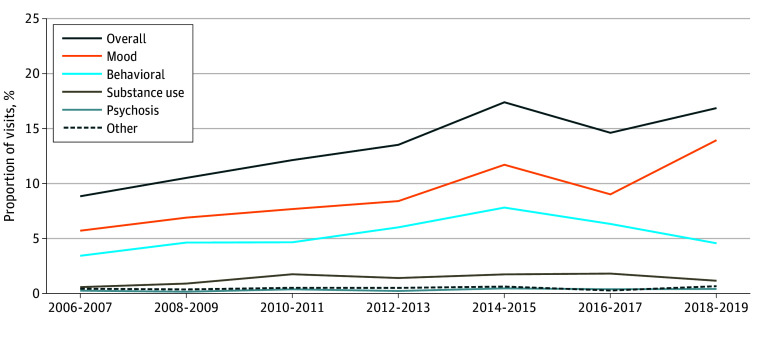
Prevalence of Mental Health–Related Outpatient Visits Among Adolescents and Young Adults Significant increases were seen for overall visits (*P* < .001), mood-related visits (5.7% to 14.0%; *P* < .001), behavioral visits (3.4% to 4.6%; *P* = .004), and substance use–related visits (0.6% to 1.2%; *P* = .04). No significant temporal changes were seen for psychosis or other visits.

The proportion of visits associated with any mental health diagnosis nearly doubled over the study period, from 8.9% in 2006 to 16.9% in 2019 (*P* < .001) (Figure 1). There were significant increases specifically for visits related to mood disorders (from 5.7% to 14.0%; *P* < .001), behavioral conditions (from 3.4% to 4.6%; *P* = .004), and substance use (from 0.6% to 1.2%; *P* = .04). Visits for mood disorders peaked in 2018-2019 for both adolescents (13.5%) and young adults (14.2%) (eFigure 1 in Supplement 1). For behavioral conditions, visits peaked in 2014-2015 at 10.2% for adolescents and 6.0% for young adults. Visits for substance use also peaked in 2014-2015 for adolescents at 0.8%, while young adults experienced a peak in 2016-2017 at 3.0%.

Increases in overall mental health–related visits were similar for female and male patients, although males had a greater burden of psychiatric illness overall (eFigure 2 in Supplement 1). Significant increases in mood-related visits were seen in both sexes (*P* < .001), with peaks of 12.9% for females and 15.8% for males in 2018-2019 (eFigure 3 in Supplement 1). Visits for behavioral disorders remained relatively constant for males, averaging 8.3% over the study period, and increased significantly for females (*P* = .002), peaking in 2014-2015 at 6.3%. There were no significant sex-based temporal changes in substance use–related visits, with averages of 0.9% for female patients and 2.0% for male patients.

### Outpatient Visits Associated With Psychotropic Medications

**Figure 2.**
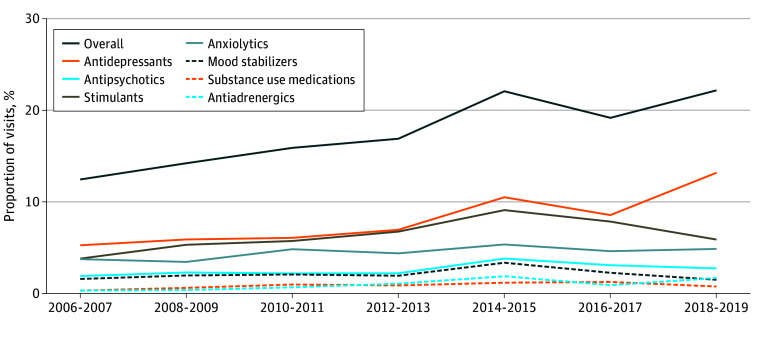
Prevalence of Mental Health–Related Outpatient Visits Among Adolescents and Young Adults by Sex Significant increases were seen for both sexes.

Among all outpatient visits for adolescents and young adults, 17.2% were associated with the prescription of at least 1 psychotropic medication, and 6.6% with 2 or more. Antidepressants were the most commonly prescribed medication class (7.8% of all visits), followed by stimulants (6.2%), anxiolytics (4.4%), antipsychotics (2.5%), and mood stabilizers (2.1%). The percentage of visits associated with the prescription of a psychotropic medication increased significantly over the study period, from 12.8% in 2006 to 22.4% in 2019 (*P* < .001) (Figure 2).

Among visits associated with a mental health diagnosis, medication use was highest for visits with behavioral conditions (84.5%), mood disorders (76.2%), and substance use (74.0%) (Table 3). Among visits by adolescent patients, males were prescribed psychiatric medications more frequently than females (79.8% vs 72.7%; *P* = .047). There were no sex-based differences in overall medication prescribing among young adult patients. When examining specific psychiatric diagnosis categories, visits by young adult females with behavioral disorders were associated with higher rates of psychotropic medication use compared with those by young adult males (90.8% vs 82.4%; *P* = .007). In addition, visits by adolescent males with other diagnoses were associated with higher rates of psychotropic medication prescribing compared with those by females (82.8% vs 50.6%; *P* < .001).

**Table 3.** Psychotropic Prescriptions Among Mental Health–Related Outpatient Visits

Diagnosis type	No. (%)			*P* value
	All visits (n = 1 105 023 336)	Visits by female patients (n = 681 918 700)	Visits by male patients (n = 423 104 636)	
Overall	111 281 136 (76.7)	55 429 764 (74.9)	55 851 372 (78.6)	.10
13-17 y	47 799 164 (76.2)	22 646 150 (72.7)	25 153 013 (79.8)	.047
18-24 y	63 481 973 (77.1)	32 783 614 (76.6)	30 698 359 (77.7)	.70
Mood	74 531 057 (76.2)	42 113 604 (75.0)	32 417 454 (77.8)	.35
13-17 y	28 594 331 (75.0)	16 615 318 (73.4)	11 979 014 (79.4)	.22
18-24 y	45 936 726 (77.8)	25 498 286 (76.0)	20 438 440 (77.0)	.80
Behavioral	48 872 751 (84.5)	19 390 868 (85.6)	29 481 883 (83.8)	.47
13-17 y	29 213 309 (83.5)	10 383 449 (81.5)	18 829 860 (84.6)	.39
18-24 y	19 659 442 (86.1)	9 007 419 (90.8)	10 652 023 (82.4)	.007
Psychosis	2 734 259 (77.3)	592 280 (64.2)	2 141 979 (82.0)	.04
13-17 y	608 867 (63.5)^a^	103 686 (29.3)^a^	505 180 (83.5)^a^	.02
18-24 y	2 125 392 (82.5)	488 594 (85.9)	1 636 798 (81.5)	.76
Substance use	10 598 279 (74.0)	3 915 490 (67.0)	6 682 789 (78.8)	.05
13-17 y	1 007 763 (41.5)	460 694 (33.9)	547 068 (51.1)	.27
18-24 y	9 590 516 (80.6)	3 454 796 (77.0)	6 135 720 (82.8)	.33
Other	3 563 770 (64.8)	1 974 988 (56.1)	1 588 782 (80.2)	.01
13-17 y	1 491 741 (63.0)	737 099 (50.6)	754 642 (82.8)	<.001
18-24 y	2 072 030 (66.1)	1 237 889 (56.0)	834 140 (78.0)	.27

^a^ Per the National Center for Health Statistics, these estimates are considered unreliable since they are based on a sample size of <30 visits.

## Discussion

The findings of our cross-sectional study suggest that the proportion of outpatient visits for mental health–related conditions increased significantly among adolescents and young adults from 2006 to 2019. This rise was associated with increases in visits for mood, behavioral, and substance use–related conditions. Mental health–related diagnoses were more prevalent among visits by male patients, particularly among young adults. Trends in prescribing of psychotropic drugs mirrored increases in mental health–related outpatient visits, with the greatest increases seen in visits associated with antidepressants.

The annual proportion of mental health–related outpatient visits increased almost 2-fold over the study period. Our findings suggest a continuation of trends seen in earlier studies documenting increases in pediatric outpatient visits associated with psychiatric illness from 1996 to 2012.^9,10^ In addition, our findings are consistent with increases in the burden of mental health conditions seen in other settings, including visits to emergency departments and hospitalizations for psychiatric conditions.^6,7,26^ These trends are likely predominately related to changing prevalence of underlying psychiatric illness in the US population,^27,28^ although a combination of other factors, including increased recognition and detection of mental illness, expanded access to outpatient care, and increase in help-seeking behavior in the setting of reduced stigmatization of mental illness may be contributing to these patterns.

There were significant differences in the rates of visits for mental health conditions based on patient sex. Males carried a greater burden of psychiatric illness, with a higher prevalence of mood, behavioral, psychosis, and substance use disorders compared with females. This aligns with condition-specific studies reporting increased prevalence of behavioral, psychosis, and substance-related conditions among males.^29,30,31^ These sex-specific findings have been attributed to differences in underlying biological factors, timing of emergence of disease (females have later onset of psychotic disorders than males), manifestation of illness (eg, conduct disorder), and socialization. One unexpected finding, however, was the increased prevalence of mood disorders among young adult males. Consistent with prior studies, adolescent visits with mood-related disorders were more common among females.^32,33^ However, this trend reversed among young adult males who had approximately twice the prevalence of mood disorders. The reason for this is unclear and in contrast to prior studies.^27,34,35^ Non–US-based studies, including one in Norway and one examining global disease trends, reported a greater burden of mood disorders among females than males in their twenties.^34,35^ However, a study of young adults in the US found no sex-based differences in rates of depression in the young adult population.^27^ It is generally accepted that across the life span, females have a greater prevalence of mood disorders than males.^36,37^ However, the transitional period from youth to adulthood presents unique challenges for males because of gender norms around masculinity, avoidance in seeking mental health services, increased exposure to violence, higher levels of substance use, and homelessness.^38,39^ Other contributing factors may be related to treatment effects of stimulants, which may lead to psychotic, depressive, and/or anxiety symptoms, and an increase in subthreshold psychiatric diagnoses, although it is unclear whether this is occurring disproportionately among males.^40,41,42^ Additional studies examining the potential association of these factors with the prevalence of specific mental health conditions in young adult males are needed.

Consistent with the rising proportion of visits for mental health conditions, we observed an increase in the proportion of outpatient visits associated with psychotropic prescribing over the study period. Nearly one-quarter of all outpatient visits were associated with a psychotropic medication in 2019. Our findings extend the results of previous work that showed increasing trends in psychotropic prescriptions among adolescents from 1994 through 2001.^43^ Several possible factors may have contributed to these trends, including increased prevalence of mental health conditions in recent years, increased severity of illness requiring pharmacologic treatment, limited accessibility to psychotherapy,^44^ and new psychotropic medication options. We were not able to assess whether changing trends were the result of increased access to psychiatric care with appropriate treatment of rising mental health conditions or whether the increases were reflective of an overreliance on medications with underuse of nonpharmacologic treatments, such as psychotherapy, exercise, and dietary changes. Irrespective of the underlying factors, use of psychotropic drugs in adolescents in particular requires careful assessment of the risk-benefit balance given the limited data on efficacy of these drugs in the pediatric population and known adverse effects, including concerns of suicidality among adolescents treated with antidepressants.^45,46,47,48^

Concerns about youth mental health remain elevated 3 years after the onset of the COVID-19 pandemic, with multiple studies documenting the negative influence of the pandemic on the mental health of adolescents and young adults.^49,50^ Our study provides additional context to the current mental health crisis, indicating that substantial increases in mental health conditions were occurring already for a prolonged period before the pandemic. This suggests that the high burden of mental health conditions documented since the onset of the pandemic cannot be attributed to the effects of this event alone and solutions will need to account for underlying factors predating the pandemic. In addition, while there has been a focus on the decline of female adolescent mental health related to the pandemic, our study points to young adult males as another potentially vulnerable population.^32,51^

### Limitations

This study has several limitations. First, NAMCS samples visits rather than patients, and therefore there may be repeated outpatient visits by the same patient, potentially inflating the estimated prevalence of mental health conditions and psychotropic prescriptions. However, this is unlikely to have substantially impacted our results given the large number of visits sampled over geographically dispersed sites. Second, mental health–related visits were identified based on assigned diagnoses, which may not always be comprehensive or represent the principal reason for a health care encounter. Third, estimates from before and after 2016 may be prone to bias due to differences between *ICD-9-CM* and *ICD-10-CM* codes. Fourth, medication information consisted of prescriptions provided and may not correspond to prescriptions filled or administered. Fifth, surveys are limited to office-based practice, so our results may not be generalizable to other treatment settings where adolescents and young adults receive mental health care, including emergency departments, inpatient settings, residential programs, and hospital-affiliated outpatient clinics.

## Conclusions

The findings of our cross-sectional study suggest substantial increases in mental health–related outpatient visits and use of psychotropic medications among adolescents and young adults from 2006 to 2019. Psychiatric illnesses were significantly more prevalent among visits by males, particularly among young adults. In the context of the current mental health crisis, these findings suggest that increases in mental health conditions seen among youth during the pandemic occurred in the setting of already increasing rates of psychiatric illness, and treatment and prevention strategies will need to account for factors beyond the direct and indirect effects of the pandemic.

## Notes

Supplement 1. **eTable 1.** *ICD* Codes for Mental Health–Related Outpatient Visits **eTable 2.** Classes of Psychotropic Medications **eFigure 1.** Age-Stratified Trends in Visits for Mood, Behavioral, and Substance-Use Disorder **eFigure 2.** Sex-Stratified Trends in Mood, Behavioral, and Substance-Use Disorders **eFigure 3.** Prevalence of Mental Health–Related Outpatient Visits Among Adolescents and Young Adults by Sex

Supplement 2.
Data Sharing Statement
