# Errors in Abstract, Methods, Figure 2, and Supplement 1

**DOI:** 10.1001/jamanetworkopen.2024.10104

**Published:** 2024-04-04

In the Original Investigation titled “Mental Health–Related Outpatient Visits Among Adolescents and Young Adults, 2006-2019,”^1^ published March 7, 2024, there were errors in the Abstract, Methods, Figure 2, and Supplement 1. In the Abstract, the age range for adolescents given as “12-17 years” should have been “13-17 years.” In Methods, “up to 30 from 2013 to 2019” should have been “up to 30 from 2014 to 2019.” In Figure 2, the title and caption were inaccurate. In Supplement 1, original eFigure 2 should have been eFigure 3 and vice versa. This article has been corrected.^1^
