# Supplementary material for: Mental Health–Related Outpatient Visits Among Adolescents and Young Adults, 2006-2019
Source: JAMA Netw Open. 2024 Mar 7;7(3):e241468. doi: 10.1001/jamanetworkopen.2024.1468 (PMC10921253; doi:10.1001/jamanetworkopen.2024.1468)
Supplement: Supplement 1. — eTable 1. ICD Codes for Mental Health–Related Outpatient Visits eTable 2. Classes of Psychotropic Medications eFigure 1. Age-Stratified Trends in Visits for Mood, Behavioral, and Substance-Use Disorder eFigure 2. Sex-Stratified Trends in Mood, Behavioral, and Substance-Use Disorders eFigure 3. Prevalence of Mental Health–Related Outpatient Visits Among Adolescents and Young Adults by Sex [file jamanetwopen-e241468-s001.pdf]

Supplementary Online Content

Ahn-Horst RY, Bourgeois FT. Mental health–related outpatient visits among adolescents and young adults, 2006-2019. *JAMA Netw Open*. 2024;7(3):e241468. doi:10.1001/jamanetworkopen.2024.1468

eTable 1. *ICD* Codes for Mental Health–Related Outpatient Visits
eTable 2. Classes of Psychotropic Medications
eFigure 1. Age-Stratified Trends in Visits for Mood, Behavioral, and Substance Use Disorders
eFigure 2. Sex-Stratified Trends in Mood, Behavioral, and Substance Use Disorders
eFigure 3. Prevalence of Mental Health–Related Outpatient Visits Among Adolescents and Young Adults by Sex

This supplementary material has been provided by the authors to give readers additional information about their work.

© 2024 Ahn-Horst RY et al. *JAMA Network Open.*

eTable 1. *ICD* Codes for Mental Health-Related Outpatient Visits

	**ICD9**	**ICD10**
**Mood**	296.XX; 300.00; 300.01; 300.02; 300.09; 300.10; 300.11; 300.12; 300.13; 300.14; 300.15; 300.20; 300.21; 300.22; 300.23; 300.29; 300.3; 300.4; 300.5; 300.6; 300.7; 300.81; 300.82; 300.89; 300.9; 308.XX; 309.0; 309.1; 309.21; 309.24; 309.28; 309.29; 309.3; 309.4; 309.81; 309.89; 309.99; 311; 313.0; 313.23; 306.XX	F30.XX; F31.XX; F32.XX; F33.XX; F34.XX; F40.XX; F41.XX; F42.XX; F43.0; F43.10; F43.11; F43.12; F43.20; F43.21; F43.22; F43.23; F44.XX; F45.XX; F48.XX; F93.XX; F94.0; O90.6
**Behavioral**	312.XX; 313.81; 314.00; 314.01; 314.2; 314.9	F43.24; F43.25; F63.XX; F90.XX; F91.XX; F95.XX
**Substance Use**	291.XX; 292.0; 292.11; 292.12; 292.2; 292.81; 292.83; 292.84; 292.85; 292.89; 292.9; 303.XX; 304.XX; 305.XX; V65.42	F10.XX; F11.XX; F12.XX; F13.XX; F14.XX; F15.XX; F16.XX; F17.XX; F18.XX; F19.XX; T40.XX; T41.XX; T42.XX
**Psychosis**	295.XX; 297.XX; 298.XX	F20.XX; F21.XX; F22.XX; F23.XX; F25.XX; F28.XX; F29.XX; F53.1
**Suicidal Behavior**	V96.84; E95.XX	R45.851; T14.91; X71-X83; T36-T65*
**Other**	301.XX; 302.0; 302.6; 302.85; 307.1; 307.20; 307.21; 307.22; 307.23; 307.50; 307.51; 307.52; 307.59; 307.80; 307.89; V61.0; V61.01; V61.02; V61.03; V61.04; V61.05; V61.06; V61.07; V61.08; V61.09; V61.1; V61.10; V61.11; V61.12; V61.2; V61.20; V61.21; V61.22; V61.23; V61.24; V61.25; V61.29; V70.1; V70.2	F50.00; F50.01; F50.02; F50.2; F50.8; F50.81; F50.89; F51.82; F51.89; F50.9; F60.XX; F69; F95.XX; F98.0; F98.1; F98.21; F98.29; F98.3; F98.9; F99

© 2024 Ahn-Horst RY et al. *JAMA Network Open.*

eTable 2. Classes of Psychotropic Medications

**Antidepressants**	**Antipsychotics**	**Stimulants**	**Anxiolytics**	**Mood Stabilizers**	**Treatment for Substance Use**	**Antiadrenergics**
Amitriptyline	Aripiprazole	Amphetamine	Alprazolam	Carbamazepine	Acamprosate	Clonidine
Bupropion	Asenapine	Amphetamine-dextroamphetamine	Buspirone	Lamotrigine	Buprenorphine	Guanfacine
Citalopram	Brexpiprazole	Atomoxetine	Butabarbital	Lithium	Buprenorphine-Naloxone	
Clomipramine	Chlorpromazine	Dexmethylphenidate	Chlordiazepoxide	Valproic acid	Disulfiram	
Desipramine	Clozapine	Dextroamphetamine	Clonazepam	Divalproex	Gabapentin	
Desvenlafaxine	Fluphenazine	Lisdexamfetamine	Clorazepate	Oxcarbazepine	Methadone	
Doxepine	Haloperidol	Methylphenidate	Diazepam	Topiramate	Naltrexone	
Duloxetine	Iloperidone	Modafinil	Diphenhydramine		Nicotine	
Escitalopram	Loxapine	Phentermine	Doxylamine		Varenicline	
Fluoxetine	Lurasidone	Phendimetrazine	Estazolam			
Fluvoxamine	Olanzapine		Hydroxyzine			
Imipramine	Paliperidone		Lorazepam			
Levomilnacipran	Perphenazine		Midazolam			
Milnacipran	Pimozide		Oxazepam			
Mirtazapine	Prochlorperazine		Phenobarbital			
Nefazodone	Promazine		Promethazine			
Nortriptyline	Quetiapine		Temazepam			
Paroxetine	Risperidone		Triazolam			
Rasagiline	Thioridazine		Zaleplon			
Selegiline	Thiothixene		Zolpidem			
Sertraline	Trifluoperazine					
Trazodone	Ziprasidone					
Vilazodone	Droperidol					
Vortioxetine						
Venlafaxine						

© 2024 Ahn-Horst RY et al. *JAMA Network Open.*

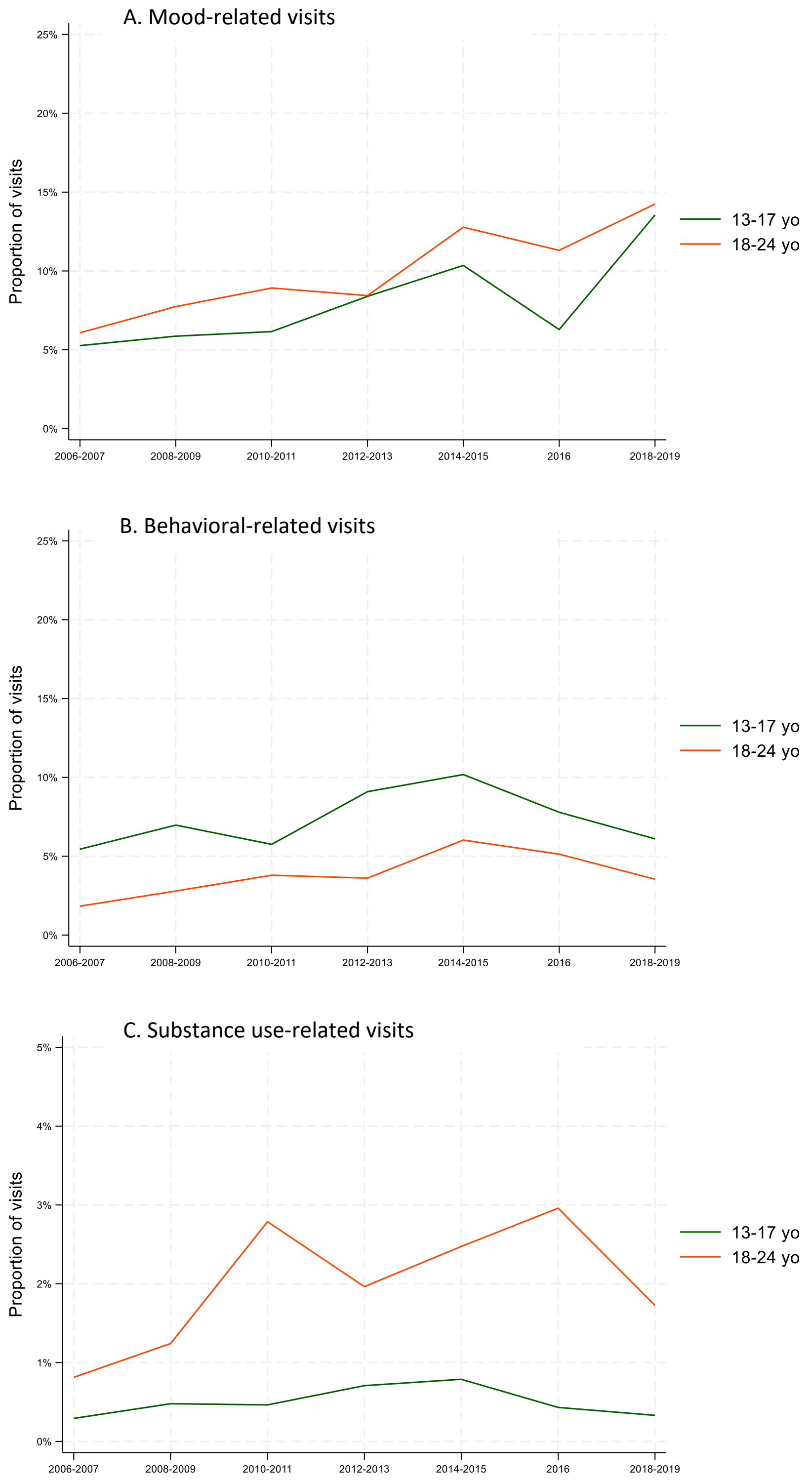

eFigure 1. Age-stratified trends in visits for mood, behavioral, and substance use disorders
Significant linear increases were seen for mood-related visits among both adolescents and young adults (p<0.001) (Panel A). Significant linear increases were also observed in behavioral-related visits among young adults (p=0.001), but not for adolescents (p=0.10) (Panel B). There were no significant linear changes for substance use-related visits among adolescents (p=0.52) or young adults (p=0.08) (Panel C).

© 2024 Ahn-Horst RY et al. *JAMA Network Open.*

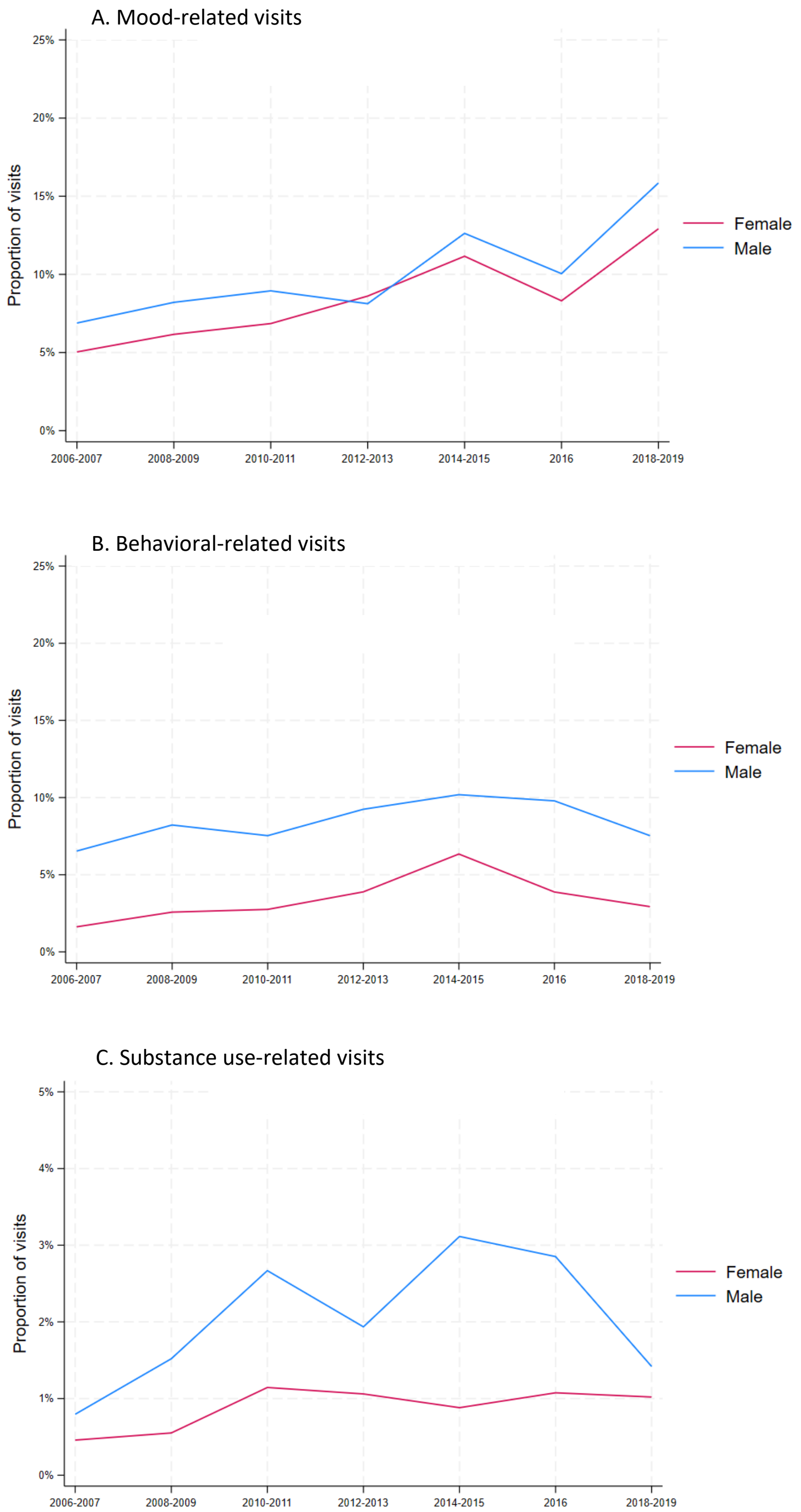

eFigure 2. Sex-stratified trends in mood, behavioral, and substance use disorders
Significant linear increases were seen for mood-related visits in both females and males ($p<0.001$) (Panel A). Significant linear increases were also seen in behavorial-related visits among females ($p=0.002$), but not among males ($p=0.17$) (Panel B). No significant linear changes were seen in substance use-related visits among females ($p=0.15$) or males ($p=0.17$) (Panel C).

© 2024 Ahn-Horst RY et al. *JAMA Network Open.*

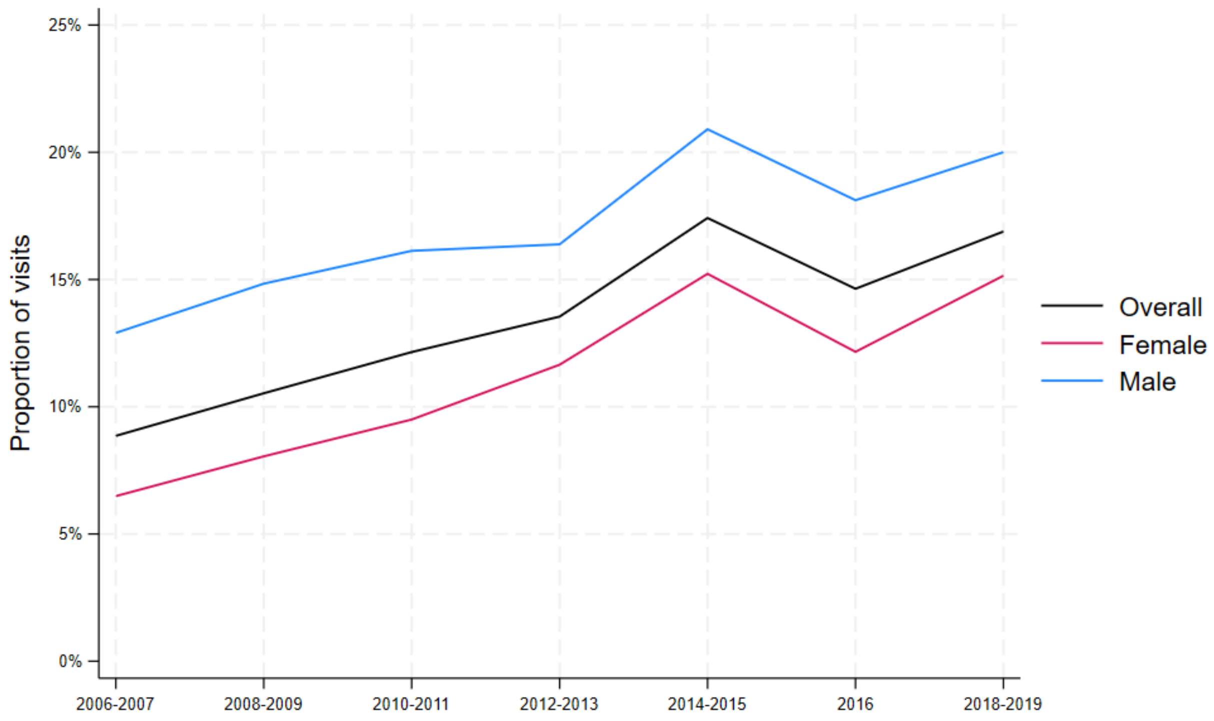

eFigure 3. Prevalence of mental health-related outpatient visits among adolescents and young adults by sex
Significant increases were seen for both sexes ($p<0.001$).

© 2024 Ahn-Horst RY et al. *JAMA Network Open.*
