# Supplementary material for: Mental Health–Related Outpatient Visits Among Adolescents and Young Adults, 2006-2019
Source: JAMA Netw Open. 2024 Mar 7;7(3):e241468. doi: 10.1001/jamanetworkopen.2024.1468 (PMC10921253; doi:10.1001/jamanetworkopen.2024.1468)
Supplement: Supplement 2. — Data Sharing Statement [file jamanetwopen-e241468-s002.pdf]

Data Sharing Statement

Ahn-Horst. Mental Health–Related Outpatient Visits Among Adolescents and Young Adults, 2006-2019. *JAMA Netw Open*. Published March 07, 2024.
doi:10.1001/jamanetworkopen.2024.1468

Data
Data available: Yes
Data types: Deidentified participant data
How to access data: All data used in this study are publicly available through the National Center for Health Statistics at https://www.cdc.gov/nchs/ahcd/index.htm.
When available: With publication

Supporting Documents
Document types: None

Additional Information
Who can access the data: Anyone interested in using the data.
Types of analyses: Any analyses per NCHS guidelines
Mechanisms of data availability: Data can be downloaded from the website at https://www.cdc.gov/nchs/ahcd/index.htm
